# DDX24 promotes metastasis by regulating RPL5 in non‐small cell lung cancer

**DOI:** 10.1002/cam4.4835

**Published:** 2022-07-21

**Authors:** Xinyan Hu, Fangfang Li, Yulan Zhou, Hairun Gan, Tiancheng Wang, Luting Li, Haoyu Long, Bing Li, Pengfei Pang

**Affiliations:** ^1^ Department of Interventional Medicine The Fifth Affiliated Hospital, Sun Yat‐sen University Zhuhai P.R. China; ^2^ Guangdong Provincial Key Laboratory of Biomedical Imaging The Fifth Affiliated Hospital, Sun Yat‐sen University Zhuhai P.R. China; ^3^ Guangdong Provincial Engineering Research Center of Molecular Imaging The Fifth Affiliated Hospital, Sun Yat‐sen University Zhuhai P.R. China; ^4^ Institute of Interventional Radiology Sun Yat‐Sen University Zhuhai P.R. China; ^5^ Beijing Institute of Brain Disorders, Laboratory of Brain Disorders, Ministry of Science and Technology, Collaborative Innovation Center for Brain Disorders Capital Medical University Beijing P.R. China; ^6^ Department of Nursing The Fifth Affiliated Hospital, Sun Yat‐sen University Zhuhai P.R. China; ^7^ Department of Ophthalmology The Fifth Affiliated Hospital, Sun Yat‐sen University Zhuhai P.R. China

**Keywords:** DDX24, metastasis, non‐small cell lung cancer, RPL5, ubiquitination

## Abstract

**Purpose:**

Non‐small cell lung cancer (NSCLC) is a leading cause of cancer death, and metastasis is a crucial determinant of increased cancer mortality. DDX24 has garnered increased attention due to its correlation with tumorigenesis and malignant progression. However, the correlation between DDX24 and NSCLC remains unclear.

**Methods:**

DDX24 expression in NSCLC tissues and survival rate of patients was analyzed using bioinformatic analysis. Transwell assays, wound‐healing assays, and tail vein lung colonization models were employed to determine the role of DDX24 in migration and invasion in vitro and in vivo. We searched for DDX24‐interacting proteins using co‐immunoprecipitation followed by mass spectroscopy and verified the interaction. The influence of DDX24 on RPL5 expression and ubiquitination was examined using protein stability assays.

**Results:**

DDX24 expression was upregulated in NSCLC cell lines and tumors of patients, particularly those with high tumor grades. A high DDX24 level was also correlated with a poor prognosis. DDX24 upregulation enhanced the migration and invasion ability of NSCLC cells, whereas its downregulation had the opposite effects. In vivo xenograft experiments confirmed that tumors with high DDX24 expression had higher metastatic abilities. The interaction between DDX24 and RPL5 promoted its ubiquitination and destabilized it.

**Conclusions:**

DDX24 acted as a pro‐tumorigenic factor and promoted metastasis in NSCLC. DDX24 interacted with RPL5 to promote its ubiquitination and degradation. As a result, targeting DDX24/RPL5 axis may provide a novel potential therapeutic strategy for NSCLC.

## Graphical abstract

DDX24 contributes to NSCLC metastasis and interactes with RPL5 to promote its ubiquitination and degradation.

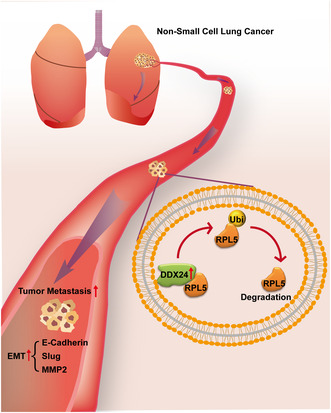

## 1 INTRODUCTION

Non‐small cell lung cancer (NSCLC) accounted for approximately 85% of all lung cancers, making it one of the most common cancers worldwide. Lung adenocarcinoma (LUAD), squamous cell carcinoma, and large cells are the most common pathological types of NSCLC.^1^ Due to the high recurrence rate and metastasis, NSCLC patients are constantly diagnosed with poor prognoses, especially at advanced disease stages. According to the World Health Organization, the 5‐year overall survival rate remains less than 5% for patients with metastatic NSCLC until the past decade, with a low overall cure rate.^2^ NSCLC treatments have progressed from cytotoxic therapies to regimens targeting specific molecular subtypes.^3^ Therefore, elucidating the possible mechanisms underlying NSCLC metastasis is critical.

*DDX24* has garnered increased attention in recent years due to its correlation with cancer development and progression. DDX24 is a family member of Asp‐Glu‐Ala‐Asp (DEAD) box containing RNA helicases, and DEAD‐box RNA helicases are characterized by a conserved DEAD motif.^4^ DEAD‐box proteins bear carboxy‐ and N‐terminal domains with high variability in sequence and length, implying diverse biological functions.^5^ The characteristic function of DEAD‐box family is to participate in RNA metabolic processes, such as RNA transcription, degradation, export, ribosomal biogenesis, and translation.^6^, ^7^ Emerging evidence has indicated that DEAD‐box family was differentially expressed in tumors and normal tissues, and DEAD‐box proteins served important roles by affecting tumor cell proliferation, metastasis, and apoptosis during cancer initiation and progression. For instance, DDX21 and DDX46 mainly function as promoters of cancer,^8^, ^9^ while DDX3 and DDX10 primarily act as inhibitors.^10^, ^11^ In previous studies, DDX24 was associated with cancer development,^12^ viral infection,^13^ and vascular malformation.^14^ However, the role of DDX24 in developing different tumors was complicated,^6^, ^15^ and the correlation between DDX24 and NSCLC remains unclear and requires further investigation.

As a component of ribosomes, ribosomal protein L5 (RPL5) is critical for regulating protein synthesis. Together with 5S rRNA and RPL11, RPL5 comprises 5S ribonucleoprotein particle (5S RNP), an assembly intermediate of a large 60S subunit of the human ribosome. 5S RPN is imported into the nucleolus and is coordinated with cell growth and proliferation.^16^ Besides, RPL5 exhibits extra ribosomal function in many cell pathological and physiological processes and has been linked to several complex disorders, such as breast cancer,^17^ hepatocellular carcinoma,^18^ multiple myeloma,^19^ and T‐cell acute lymphoblastic leukemia.^20^ RPL5 has also been linked to known prominent tumor suppressors such as p53.^21^, ^22^

This study identifies that DDX24 contributes to cell migration and invasion in NSCLC in vitro and in vivo and interacts with RPL5 to promote its ubiquitination and degradation. A better understanding of this novel mechanism of NSCLC allows more effective applications of targeted therapies to improve prognosis.

## 2 METHODS

### 2.1 Cell culture, transfection, and transduction

HFL1 (Catalog# CCL‐153), NCI‐H1299 (Catalog# CRL‐5803), NCI‐H460 (Cata‐log# HTB‐177) and A549 (Catalog# CCL‐185) were purchased from the American Type Culture Collection (ATCC). NCI‐H226 (Catalog# SCSP‐5073) and PC9 (Catalog# SCSP‐5085) were obtained from the Chinese Academy of Sciences Cell Bank (Shanghai, China). NCI‐H1299, NCI‐H460, and NCI‐H226 were cultured in Roswell Park Memorial Institute‐1640 medium (RPMI‐1640) (GIBCO, Catalog# C11875500BT) supplemented with 10% fetal bovine serum (FBS) and 5 ml of penicillin/streptomycin solution. Other cells were cultured in Dulbecco's modified Eagle medium (DMEM) (GIBCO, Catalog# C11995500BT). All cells were used in experiments at passages two to seven and cultured in a humidified atmosphere at 37°C and 5%CO_2_.

NSCLC cells were transfected with vector and *DDX24*‐CMV‐GFP‐Puro plasmid (GenePharma), small hairpin RNA (shRNA‐1, 5′‐GCAGUCAAGCUGUGGCAAATT‐3′ or shRNA‐2, 5′‐GGGAGAAACCUGUUCCCAATT‐3′) or negative control (sh‐NC) (GenePharma) using Lipofectamine 3000 Transfection Reagent (Invitrogen, Catalog# L3000015). After 48 h incubation, the treated cells were collected for western blot analysis to confirm knockdown efficiency.

DDX24 stable knockdown in cell lines was established with a lentiviral vector (U6‐sh‐*DDX24‐*EGFP‐IRES‐puromycin) purchased from GeneCopoeia. According to manufacturer's instructions, the cells were infected with recombinant lentivirus transducing units. Puromycin (1 μg/ml) was used to select stable clones for 2 weeks and harvested.

### 2.2 Western blot

The samples were lysed on ice for 30 min using cell lysis buffer (Beyotime Biotechnology, Catalog# P0013C). After centrifugation at 14,000 rpm at 4°C for 20 min, the proteins in the supernatant were collected. Following this, protein concentration was determined using a BCA kit (Beyotime Biotechnology, Catalog# P0010). Appropriate quantities of protein samples were boiled with SDS loading buffer at 100°C for 5 min. 5%–20% SDS–polyacrylamide gel electrophoresis (SDS‐PAGE) gels were utilized to separated proteins. Western blot analysis was performed according to standard procedures. The primary antibodies used in this study were as follows: Anti‐DDX24 (Bethyl Laboratories, Catalog# A300‐697A), anti‐β‐actin (CST, Catalog# 4970), anti‐Gapdh (CST, Catalog# 5174), anti‐E‐cadherin (CST, Catalog# 3195), anti‐MMP2 (CST, Catalog# 40994), anti‐Slug (CST, Catalog# 9585), anti‐RPL5 (CST, Catalog# 14568S). All primary antibodies were diluted 1000‐fold following the manufacturer instructions.

### 2.3 Real‐time quantitative PCR

Total RNA used in this study was extracted from each cell line using Total RNA Kit I (Omega Bio‐Tek, Catalog# R6834‐02). Real‐time quantitative PCR (RT‐qPCR) for gene expression was performed using All‐in‐one TM qPCR Mix (GeneCopoeia TM) and Bio‐Rad CFX96 (Bio‐Rad). Gene expression levels were normalized to the levels of Gapdh, and each sample was analyzed in triplicates. Primers for DDX24 (Catalog# Hs‐QRP‐23370), GAPDH (Catalog# Hs‐QRP‐20169) were purchased from GeneCopoeia Inc. (GeneCopoeia).

### 2.4 Transwell assays

Transwell assays were used to analyze cell migration and invasion abilities. For transwell invasion assay, upper chambers were filled with 50 μl matrigels (BD Biosciences, Catalog# 354234). 3 × 10^4^ PC9, NCI‐H1299, NCI‐H460, and A549 cells were seeded on the upper chamber of 24‐well transwell chambers (8 μm pore; corning, Catalog# 353097) with 300 μl serum‐free DMEM or RPMI‐1640. A 600 μl DMEM or RPMI‐1640 with 10% FBS was added to the lower chamber. After incubation for 24 h, the cells were fixed for 30 min, stained with 0.1% crystal violet, and photographed.

### 2.5 Bioinformatic analysis

The Kaplan–Meier plotter (www.kmplot.com) was used for prognostic analysis of the survival rate. The gene affy ID of DDX24 was 200702_s_at. Overall survival (OS) data from 82 patients (GSE19188) were analyzed with the auto best cutoff selected. All other settings used in the survival analysis were default. DDX24 gene expression levels from CPTAC were obtained from UALCAN portal (http://ualcan.path.uab.edu). We chose to explore DDX24 proteomic expression profile based on tumor grade or sample types with other quantification settings kept default. No special screening criteria was applied. A total of 111 normal samples and 111 primary LUAD patients were available. Because of the missing information, only 7 grade I patients, 59 grade II patients, and 39 grade III patients were identified for further investigation.

### 2.6 Animal studies

Four‐week‐old female SCID mice (Vital River Laboratory Animal Technology Co., Ltd) were housed under pathogen‐free conditions. A total of 15 mice were randomly divided into three groups of five mice. Stable DDX24 knockdown (sh*DDX24*‐1, sh*DDX24*‐2) or control A549 cells (sh‐NC) (3 × 10^6^ cells/100 μl phosphate‐buffered saline per mouse) were injected via tail vein. Because there was no unexpected death during the injection, no exclusions were observed for each experimental group. After 9 weeks, the mice were humanely sacrificed. Lungs of all mice were harvested and embedded in paraffin for HE staining. Tumor nodules on lung surfaces were counted.

### 2.7 Co‐immunoprecipitation

Co‐immunoprecipitation (Co‐IP) experiments were performed according to the instructions of Pierce Classic IP Kit (Thermo Scientific, Catalog# 26146) with subtle modifications. Cells were collected after transfected with Flag‐DDX24 (GenePharma) for 48 h. Cold IP lysis was added to cell pellets. After incubation, the protein supernatant was collected for protein concentration determination. Protein concentrations were adjusted to the same level. Control Agarose Resin was used to pre‐clear lysate. Every 100 μg of proteins were incubated with 1 μg anti‐Flag antibody (Sigma‐Aldrich, Catalog# F1804) overnight at 4°C under constant mixing. The immune complexes were incubated with A/G Plus Agarose for 1 h at 4°C in a rotator. The resins were washed for 8–10 times and centrifugated at 1000 × g for 1 min. Protein denaturalization was performed, followed by western blot. Co‐IP products were separated on SDS‐PAGE gels. The gels were then silver‐stained by Silver Stain Plus Kit (Bio‐Rad, Catalog# 1610449). The specific bands were excised and prepared for mass spectrometry to determine DDX24‐interacting proteins.

### 2.8 Wound‐healing assay

The cells were seeded in 6‐well plates. Confluent cell monolayers were wounded with sterilized 200 μl micropipette tips to create a cell‐free zone. A serum‐free medium was added to every plate. Images were taken at 0, 12, and 24 h later. The remaining migrated area was then quantified using Image J software (National Institutes of Health).

### 2.9 Cell viability assay

Cell viability was assayed using Cell Counting Kit‐8 (CCK‐8) (KeyGEN BioTECH). According to the provided protocol, 2–3 × 10^3^ cells were seeded in 96‐well plates per well. Once cells adhered, 10 μl of CCK8 solution and 90 μl of cell culture medium were mixed and added to each well. After incubation for 2 h, absorbance was measured at 450 nm by a spectrometer. The absorbances were also measured after 24 and 48 h of cell culture.

### 2.10 Gelatin zymography

MMP2 activity was detected using MMP Zymography Assay Kit (APPLYGEN, Catalog# P1700). Cells were seeded in 6‐well plates and cultured with 300 μl of cell culture medium without FBS for 24 h. Then, the supernatant containing secretory MMP2 was collected. According to the manufacturer's protocols, equal amounts of protein were used and separated in 8% gelatin zymogram gel. Then, the gels were incubated in renaturing solution at room temperature for 36 h and in development solution at 37°C for 5 h. Stained with 0.5% Coomassie blue R‐250 for 1 h, the gels were destained with the destaining solution until clear bands showed.

### 2.11 Statistical analysis

All data were shown as mean ± SD of at least three independent experiments. Statistical analyses were performed using the help of GraphPad Prism software (version 8.0). The differences between groups were analyzed using two Student's *t*‐test or one‐way ANOVA. Statistical significance was indicated as follows: n. s., not significant; *p < 0.05; **p < 0.01; ***p < 0.001; ****p < 0.0001.

## 3 RESULTS

### 3.1 DDX24 is upregulated in NSCLC and is correlated with poor prognosis

**FIGURE 1:**
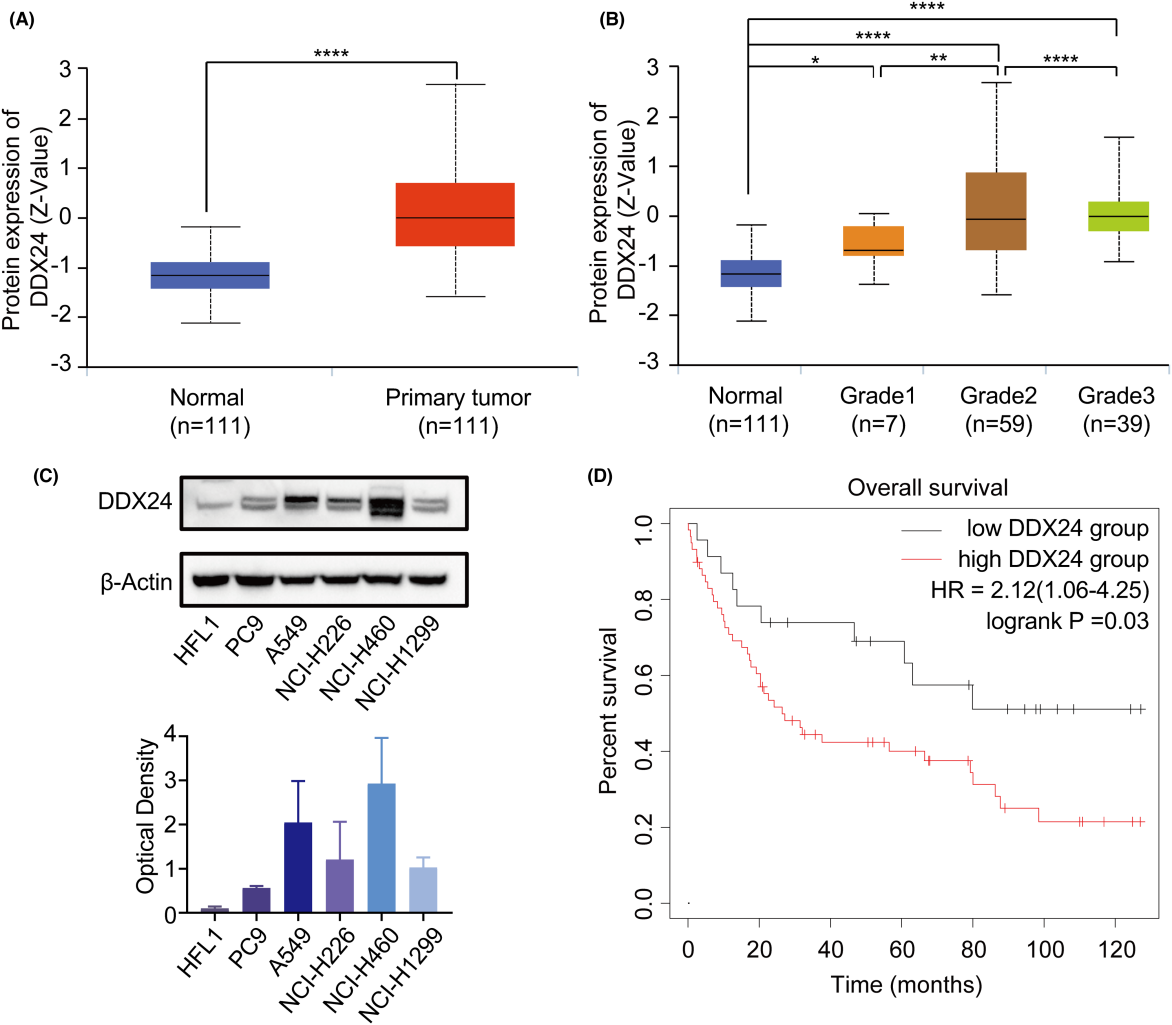
DDX24 protein is increased and is associated with poor prognosis in NSCLC patients. (A) The protein levels of DDX24 in LUAD and normal tissues from CPTAC are analyzed by UALCAN portal. (B) The protein levels of DDX24 in different grades of LUAD. (C) DDX24 protein expression in five NSCLC cell lines and human normal primary cell line HFL1 is detected using western blot (upper panel). The lower panel shows the relative protein levels quantified by gray value and normalized to β‐actin. (D) Kaplan–Meier plots of DDX24 expression in NSCLC patients. The results are represented as means ± SD. *p < 0.05; **p < 0.01; ****p < 0.0001.

First, we resorted to the clinical proteomic tumor analysis consortium (CPTAC) database to explore DDX24 expression in NSCLC. The results revealed a higher expression of DDX24 protein in LUAD than in normal tissue (Figure 1A). Intriguingly, the higher level of DDX24 protein was linked to the poor tumor grade of LUAD (Figure 1B). Further validation was conducted in a panel of NSCLC cell lines. The results validated that DDX24 was higher in NSCLC cell lines than the primary lung fibroblast cell line HFL1 (Figure 1C). We further explored the relevance of OS of patients to DDX24 expression. Based on Kaplan–Meier plotter results, high DDX24 expression was significantly correlated with poor survival (Figure 1D). These results suggest that DDX24 upregulation is strongly correlated with NSCLC progression.

### 3.2 DDX24 promotes NSCLC migration and invasion in vitro

**FIGURE 2:**
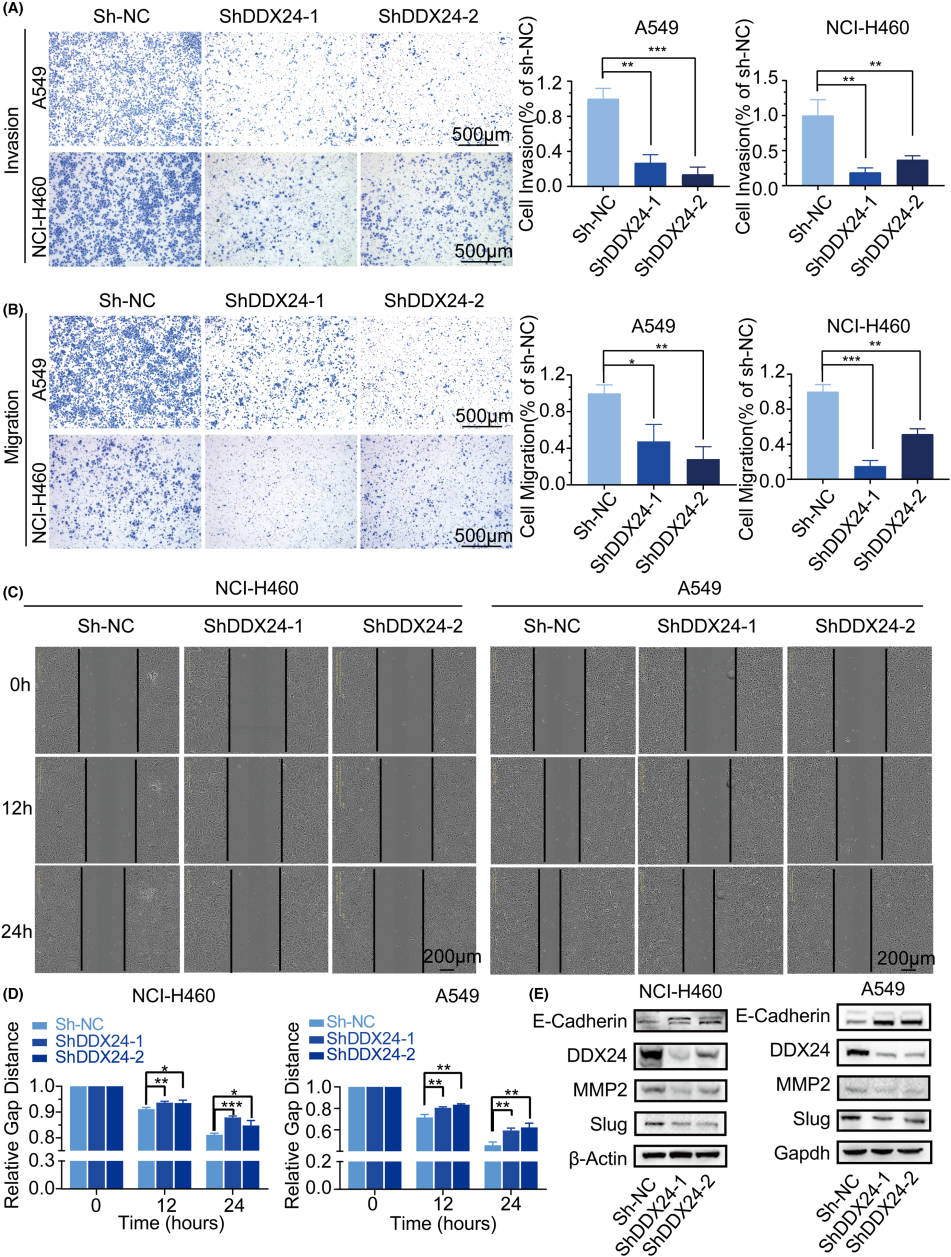
DDX24 silencing suppresses the migration and invasion of NSCLC cells. Cell invasion (A) and migration (B) in NSCLC cells with or without DDX24 knockdown are detected using transwell assay. Quantification for the number of invaded or migrated cells is displayed by the bar graph on the right. (C) Wound‐healing assay is imaged immediately, 12 and 24 h, respectively. The dashed lines demonstrate the initial margin of wound scratch. (D) The bar graph illustrates the relative scratch area at each time point. (E) The expression of EMT‐related proteins in NSCLCs with DDX24 knockdown is detected by western blot. *p < 0.05; **p < 0.01; ***p < 0.001.

**FIGURE 3:**
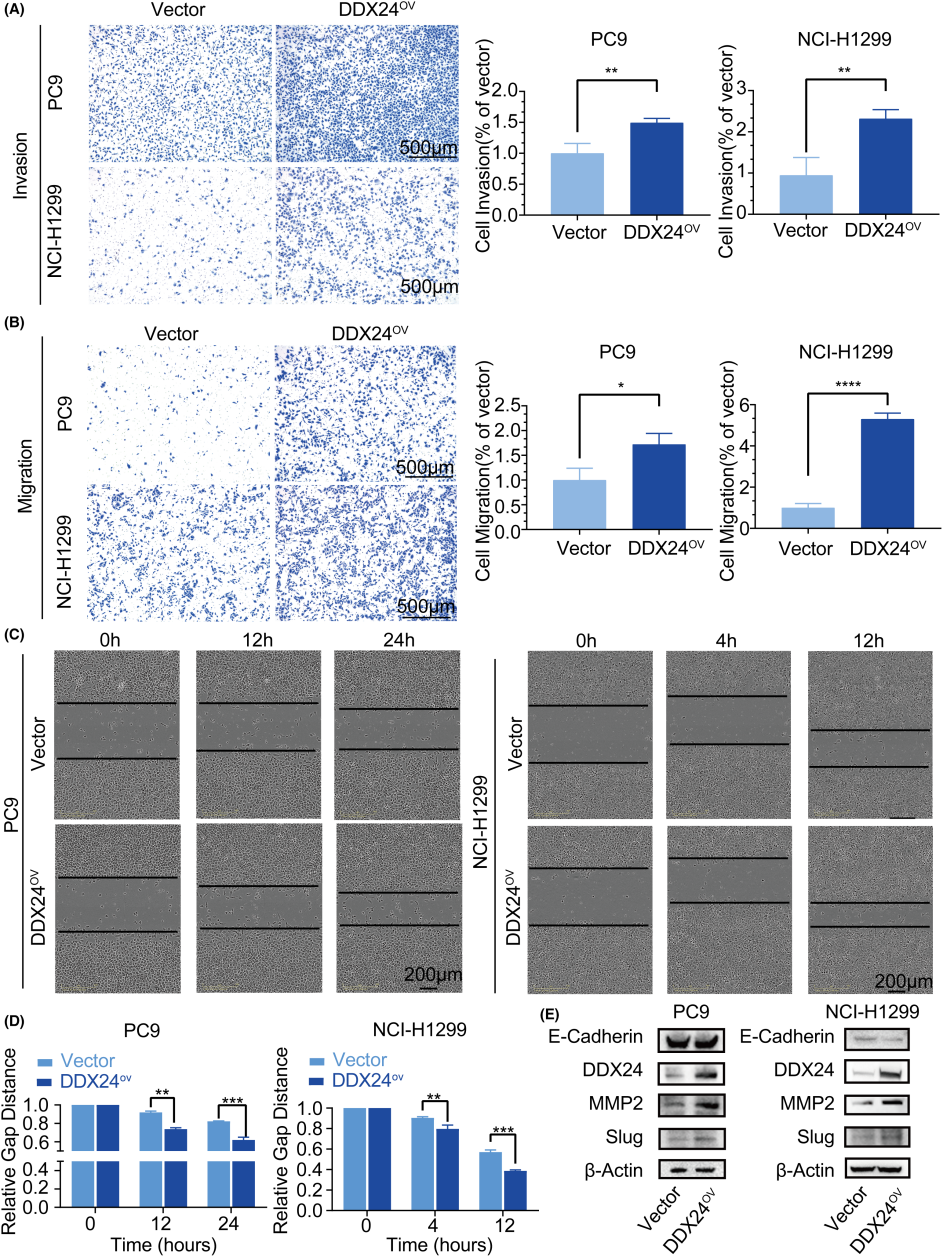
DDX24 overexpressing upregulates the migration and invasion of NSCLC cells. Cell invasion (A) and migration (B) in NSCLC cells with or without DDX24 overexpressing are explored by transwell assay. Quantification for the number of invaded or migrated cells is shown on the right. (C) Wound‐healing assay is imaged at different time points with DDX24 overexpressing. The dashed lines indicate the margin of scratches. (D) The area of the relative scratch is shown at each time point. (E) The expression of EMT‐related proteins in NSCLCs with DDX24 overexpressing is performed using western blot. *p < 0.05; **p < 0.01; ***p < 0.001; ****p < 0.0001.

Given that metastasis is an adverse progression of NSCLC‐related prognosis and OS time, we further explored the role of DDX24 in migration and invasion of NSCLC cells (Figures 2 and 3). After confirming silence or overexpression of DDX24 (Figure S1A,B), we uncovered that DDX24 reduction inhibited invasive (Figure 2A) and migratory (Figure 2B) capacity in NCI‐H460 and A549 cells. Both migration and invasion abilities of DDX24‐knockdown cells were reduced by more than 50% compared with the control group. The scratch wound assay also corroborated the effect of DDX24 silencing on decreasing cell migration (Figure 2C,D). On the contrary, DDX24 overexpression promoted invasion (Figure 3A) and migration (Figure 3B–D) in NSCLC cells. Moreover, as epithelial‐mesenchymal transition (EMT) is essential for fueling invasion,^23^ we then examined the expression of EMT‐related proteins and found that MMP2 and Slug were downregulated, whereas E‐cadherin was upregulated in DDX24 knockdown NSCLC cells (Figure 2E). MMP2 activity was also inhibited in NCI‐H460 cells with DDX24 knockdown (Figure S2A). In addition, DDX24 overexpression displayed opposite results on EMT proteins (Figure 3E). And DDX24 overexpression in NCI‐H1299 cells enhanced MMP2 activity (Figure S2B). Then, we explored the effects of DDX24 in the cell growth of NSCLC. It was found that DDX24 has little effect on the cell proliferation of NSCLC cells (Figure S3). Overall, these results indicate that DDX24 promotes migration and invasion through EMT pathways in vitro.

### 3.3 Suppression of NSCLC metastasis by DDX24 silencing in vivo

**FIGURE 4:**
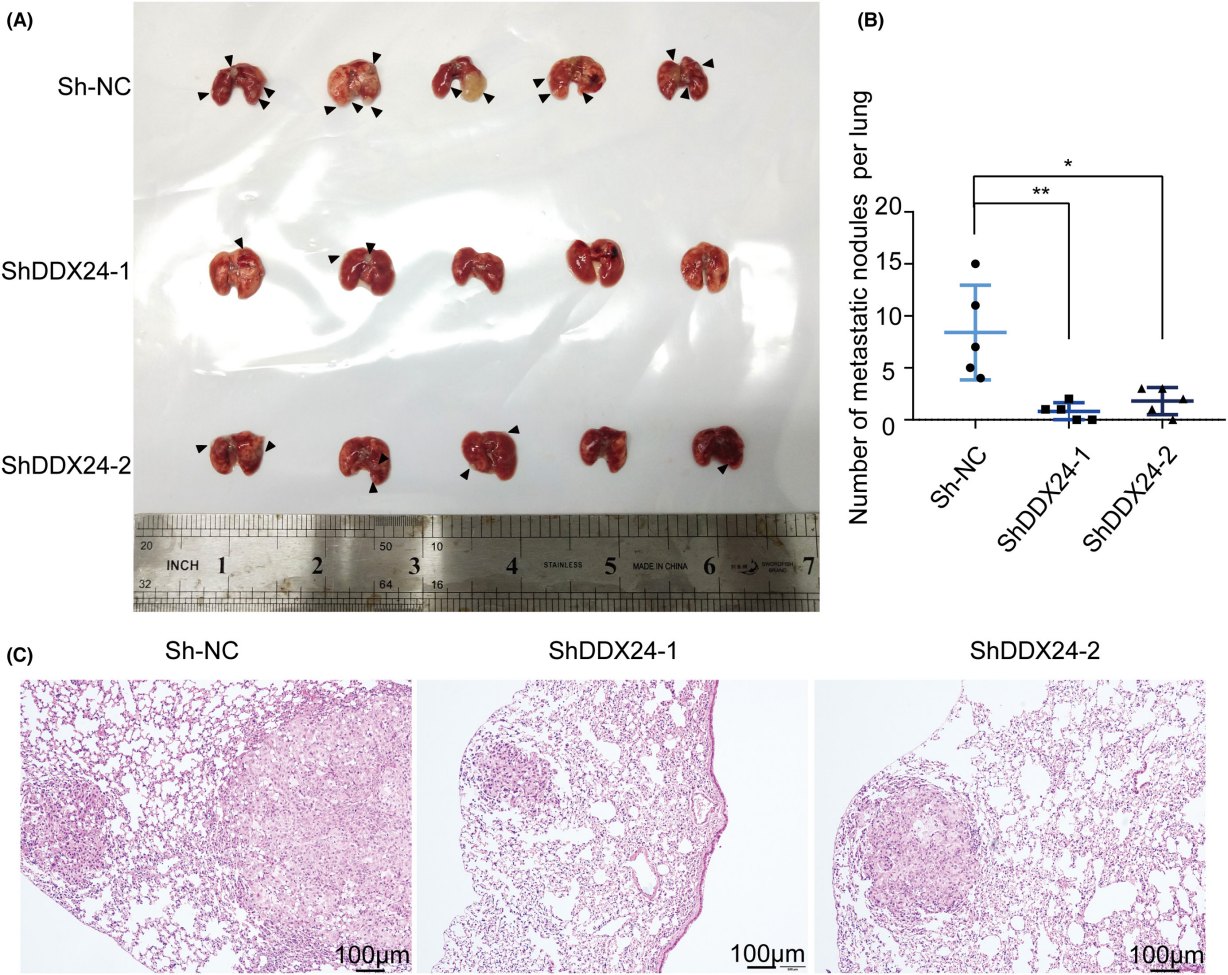
DDX24 knockdown suppresses the metastasis in vivo. (A) Representative plot of lung metastasis mouse models is shown. Each arrow pointed to a tumor lesion. (B) The amount of metastasis tumor nodules on the surface of lung tissue is calculated. (C) Metastatic tumors derived from A549 cells stably transfected with two individual shRNAs (sh*DDX24*‐1, sh*DDX24*‐2) or control shRNA (sh‐NC) are performed with HE staining. *p < 0.05; **p < 0.01.

As uncontrolled cell migration and invasion to a secondary site was the characteristic of metastasis,^24^ we next evaluated the in vivo effect of DDX24 on metastasis formation. In the malignant metastatic model, the numbers of metastasis tumor nodules observed on the surface of lungs were much less than when DDX24 was silenced (Figure 4A). The number and size of metastatic lesions in the lungs of mice with DDX24 knockdown were significantly decreased (Figure 4B), implying weak metastasis capability. HE staining further confirmed the lung metastasis (Figure 4C). These results confirm that DDX24 helps promote metastasis in vivo.

### 3.4 DDX24 promotes ubiquitination‐mediated RPL5 degradation

**FIGURE 5:**
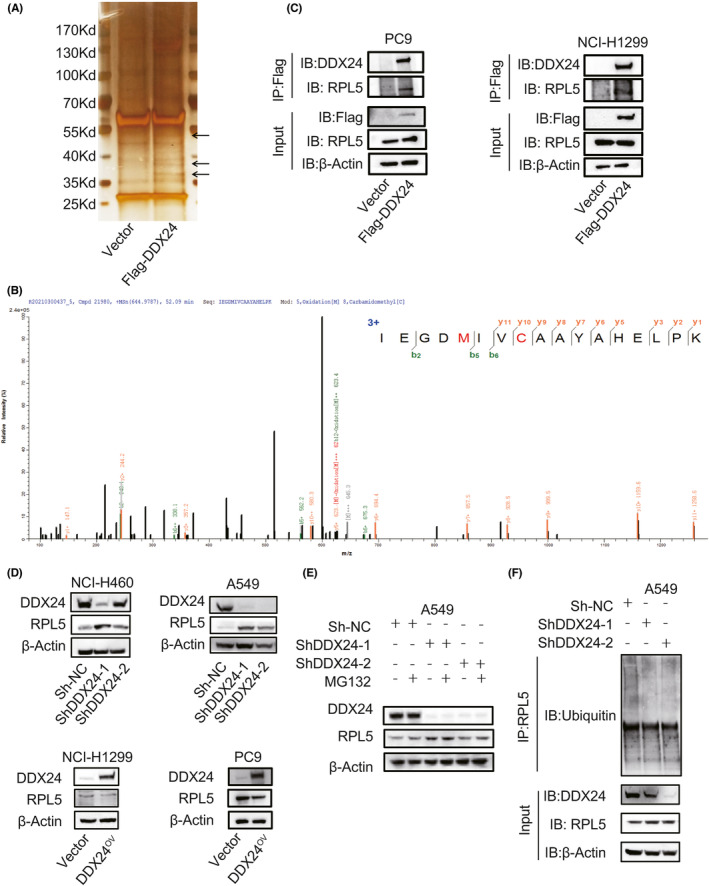
DDX24 interacts with RPL5 to regulate its ubiquitination. (A) SDS‐page gel stained with silver staining shows the extracted proteins with anti‐Flag antibody in PC9 and NCI‐H1299 cell lines. The arrows refer to the specific bands containing the candidate proteins. (B) Mass spectrometry identifies the spectra of putative proteins. (C) Co‐IP detects the extracted cell proteins with anti‐Flag antibody in the presence of Flag‐tagged DDX24 in NSCLCs. (D) Western blot is used to detect RPL5 expression with DDX24 overexpressing or silencing. (E) Western blot analysis of RPL5 is conducted after treating A549 cells with proteasome inhibitor MG132. (F) Co‐IP is performed with anti‐RPL5 antibody when DDX24 is knockdown.

To further investigate potential proteins that interacted with DDX24 to elucidate the biological mechanism of DDX24 affecting NSCLC metastasis, we conducted a co‐IP assay and mass spectrometry analysis of candidate protein bands identified 295 peptide segments (File S1). As previously described, RPL5 and DDX24 both located and worked as tumor‐related proteins within the nucleolus,^25^, ^26^ and RPL5 has a molecular mass similar to specific segments on 35 kDa in the presence of Flag‐tagged DDX24 (Figure 5A). Moreover, the assigned b and y ion peaks on the spectrum were identified as IEGDMIVCAAYAHELPK and marked with their corresponding *m/z* values (Figure 5B). Therefore, we focused on RPL5 among the candidate proteins interacting with DDX24. We confirmed that DDX24 could interact with RPL5 in NCI‐H1299 and PC9 cells (Figure 5C). Furthermore, DDX24 overexpression reduced RPL5 in NSCLC cells, while DDX24 reduction had the opposite effects (Figure 5D). Previous studies revealed that DDX24 is critical for ubiquitination regulation and protein stability.^27^, ^28^, ^29^ To examine whether DDX24 regulated the stability of RPL5 by ubiquitination, A549 cells were treated with the proteasome inhibitor MG132 (1 μM, 12 h), which resulted in a significant increase in the amount of endogenous RPL5 (Figure 5E). MG132 partly increased the expression of RPL5 in the control group. Moreover, DDX24 knockdown promoted the MG132‐induced elevation of RPL5 level, which implied that MG132 mainly interrupted the ubiquitin procession of interaction between DDX24 and RPL5. Besides, co‐IP revealed that DDX24 knockdown decreased RPL5 ubiquitination (Figure 5F). In summary, these results suggest that DDX24 promotes ubiquitination‐mediated degradation of RPL5.

## 4 DISCUSSION

Sophisticated therapies are required to fight against NSCLC, a malignancy with high incidence and lethality rates. Targeted therapy is an emerging approach for improving clinical outcomes for patients but has met with limited success in recent trials.^30^ Therefore, identifying new driver genes can maximize the benefit of targeted therapies and discover additional drug‐targetable biomarkers. Previous research revealed that DDX24 exhibits either an oncogenic or tumor‐suppressive role in different cancer types.^6^, ^15^, ^28^ Until now, the role of DDX24 in NSCLC remains largely unexamined. With the widespread use of high‐throughput technologies and the advancement in cancer‐related databases, some analyses and predictions can be performed about the function of DDX24 in NSCLC. This study disclosed that DDX24 was highly expressed in NSCLC patients, especially those with high tumor grades. Furthermore, high DDX24 expression was significantly correlated with poor survival of NSCLC patients. Accordingly, we hypothesized that DDX24 probably played an important role in NSCLC development and progression and could prolong survival with advanced tumors.

Metastasis resulted in a significant decrease in survival rate, and advanced NSCLC limited the therapeutic efficacy with metastasis.^2^ Our previous study found that DDX24 could promote vascular endothelial cell migration and tube formation. Vascular endothelial growth factor and other migration‐associated genes, such as CX3CR1 and PTAFR, were also enriched in DDX24‐knockdown cells.^14^ There are researches revealed that DDX5 and DDX3X were identified as regulators in lung cancer.^31^, ^32^, ^33^ Both vascular formation and EMT orchestrate indispensable roles in cancer metastasis.^23^, ^34^ These findings were suggestive of a correlation between DDX24 and tumor metastatic ability. In this study, in vitro experiments demonstrated that DDX24 promoted the migration and invasion ability of NSCLC cells, and in vivo xenograft experiments confirmed that NSCLC tumors with high DDX24 expression had higher metastatic abilities. The expression of EMT‐related proteins corroborated our findings. DDX24 regulates the metastasis of NSCLC cells via mediating MMP2, slug, and E‐cadherin levels. As a novel promoting factor of NSCLC metastasis, DDX24 could be a potential clinical target. DDX24 might be considered a biomarker for the early management of NSCLC, including aggressive surgery, systemic immunotherapy, and other radical therapies.

Thus far, most literature on DDX24 has emphasized that it is a multifunctional RNA helicase that participates in essential RNA metabolisms, including transcription, translation, and transport.^35^ A prior study revealed that DDX24 depletion reduced the abundance of 32S pre‐rRNA and the rate of 28S rRNA production.^28^ It is widely acknowledged that 32S, 28S, and 5S rRNAs are essential components for assembling the ribosome 60S subunit.^36^ In addition, RPL5 binds 5S rRNA to comprise a ribosomal subcomplex called 5S RNP, an assembly intermediate of a large 60S subunit.^25^ In this way, nonribosome‐associated cytoplasmic 5S rRNA is transported to the nucleolus for assembly into ribosomes.^16^ On the other side, DDX24 is accumulated predominantly within the nucleolus.^26^, ^37^ These results seem to suggest that DDX24 and RPL5 have a common pathophysiologic basis for cancer‐related regulations.

Our study confirmed these potential associations between DDX24 and RPL5. In this study, co‐immunoprecipitation followed by mass spectroscopy (IP‐MS) was employed to probe the mechanism underlying DDX24‐driving NSCLC progression. There are studies revealed that DDX24 is critical for ubiquitination regulation and protein stability. For example, the interaction between DDX24 and USP7, a ubiquitin‐specific protease, was found by affinity purification coupled with mass spectrometry.^27^ It is well known that USP7 mainly binds with specific target proteins, brings in an E3 Ub ligase or modulates the activity of the deubiquitinating enzyme which influence various cellular pathways related to cancer.^38^ In addition, Yamauchi’ study found that DDX24 can bind to the central region of MDM2, leading to the polyubiquitylation of DDX24 and then promoted its correlation with preribosomal ribonucleoprotein (pre‐rRNP) processing complexes.^28^ Therefore, we hypothesized that ubiquitination affects the interaction between DDX24 and RPL5. This hypothesis was proved, and our study uncovered that DDX24 interacted with RPL5 to promote RPL5 ubiquitination and degradation, which was a novel link between DDX24 to RPL5. It was previously observed that DDX24 depletion impaired pre‐rRNA processing and consequently inhibited MDM2 function through binding ribosomal proteins (RPs). During tumorigenesis‐associated ribosome stress, DDX24 regulated downstream of RP‐MDM2 pathway resulting in p53 stabilization.^28^ Likewise, when ribosomal assembly fails, RPL5 could respond to ribosomal biogenesis stress and sequesters MDM2, which activates p53 and contributes to a tumor suppressor activity.^39^, ^40^, ^41^, ^42^, ^43^ When these results are considered, it is possible that DDX24/RPL5 axis can link to p53, their common downstream target. Such a hypothesis requires further experimental validation.

This study exhibits some limitations. The precise role of DDX24 in NSCLC pathogenesis and treatment of diseases has not yet been well investigated. The underlying pathway by which DDX24 interacts with RPL5 and exact binding sites have yet to be elucidated. Our findings must be verified in different populations or introduced in the clinical stage.

Overall, DDX24 acts as a pro‐tumorigenic factor and promotes metastasis in NSCLC. Our study provided new insights into DDX24/RPL5 pathway that DDX24 interacts with RPL5 to promote its ubiquitination and degradation. DDX24 may serve as a potential prognostic and therapeutic target in NSCLC.

## AUTHOR CONTRIBUTIONS

The conception and design of the work were conducted by Bing Li and Pengfei Pang. Manuscript writing and revision were performed by Xinyan Hu, Fangfang Li, and Yulan Zhou. Hairun Gan and Tiancheng Wang contributed to the analysis and interpretation of data. Xinyan Hu, Luting Li, and Haoyu Long analyzed the cell and animal experiments data. All authors have approved the submitted version and agreed to be personally accountable for the author's own contributions.

## FUNDING INFORMATION

This work was supported by the National Natural Science Foundation of China (82072033); the Natural Science Foundation of Guangdong Province (2021A1515010380); the 5010 Project of Clinical Medicine Research Sun Yat‐sen University (2018011); and Science and Technology Planning Project of Guangdong Province (2020A1414010308).

## CONFLICT OF INTEREST

The authors declare that they have no competing interests.

## ETHICAL APPROVAL

This study has been performed in accordance with the principles enshrined in the Declaration of Helsinki and the Guide for the Care and Use of Laboratory Animals (Nos. 80–23, revised 1996). The work was approved and in accordance with the Animal Ethics Committee of University of Sun Yat‐sen (No.00163).

## Supporting information


Figure S1

Figure S2

Figure S3

File S1

## Data Availability

Data sharing is not applicable to this article as no new data were created or analyzed in this study.
