# Supplementary figures and images for: DDX24 promotes metastasis by regulating RPL5 in non‐small cell lung cancer
Source: Cancer Med. 2022 Jul 21;11(23):4513–25. doi: 10.1002/cam4.4835 (PMC9741967; doi:10.1002/cam4.4835)

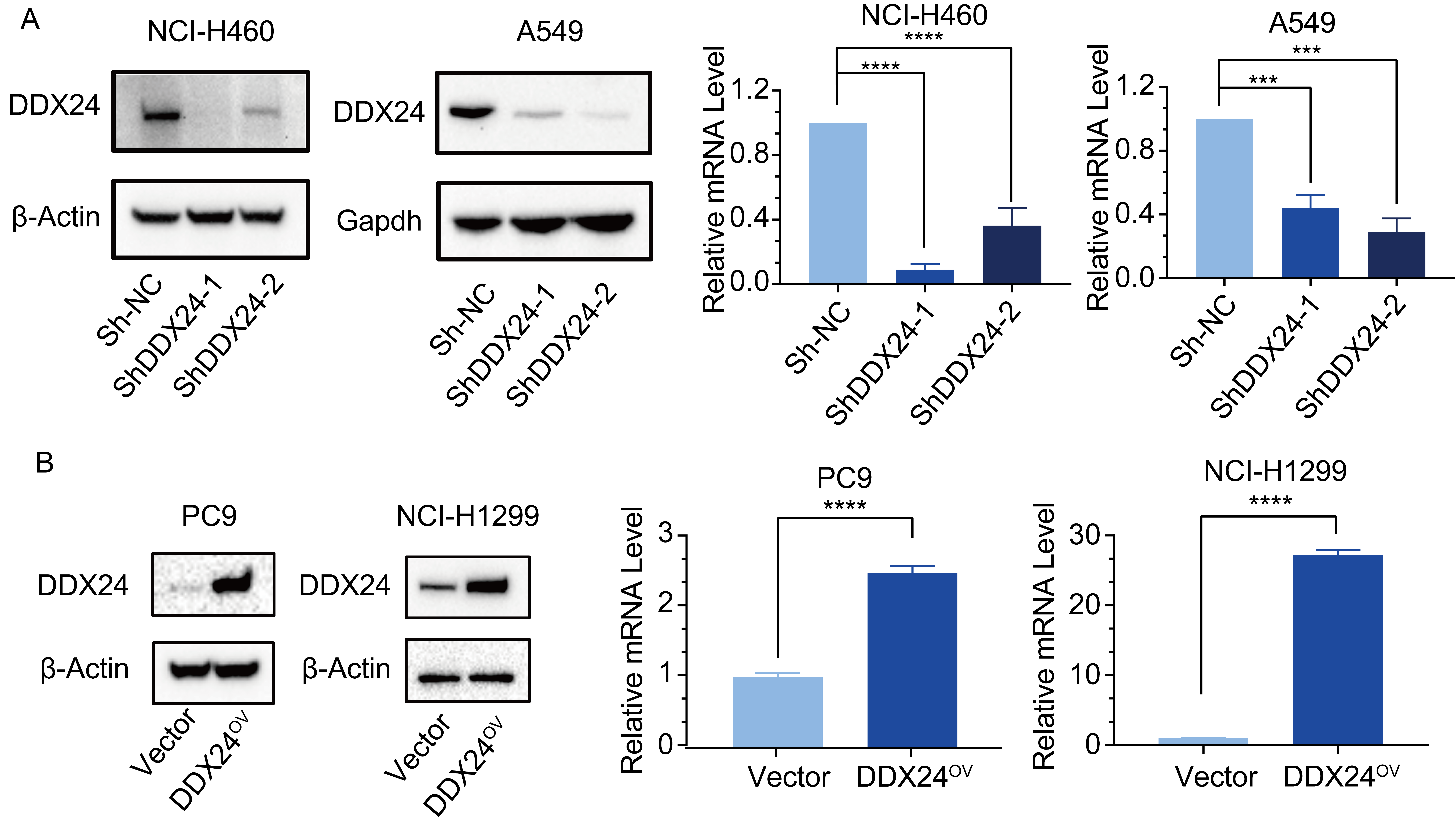

Supplement: Supplementary file 1 — Figure S1 [file CAM4-11-4513-s004.tif]

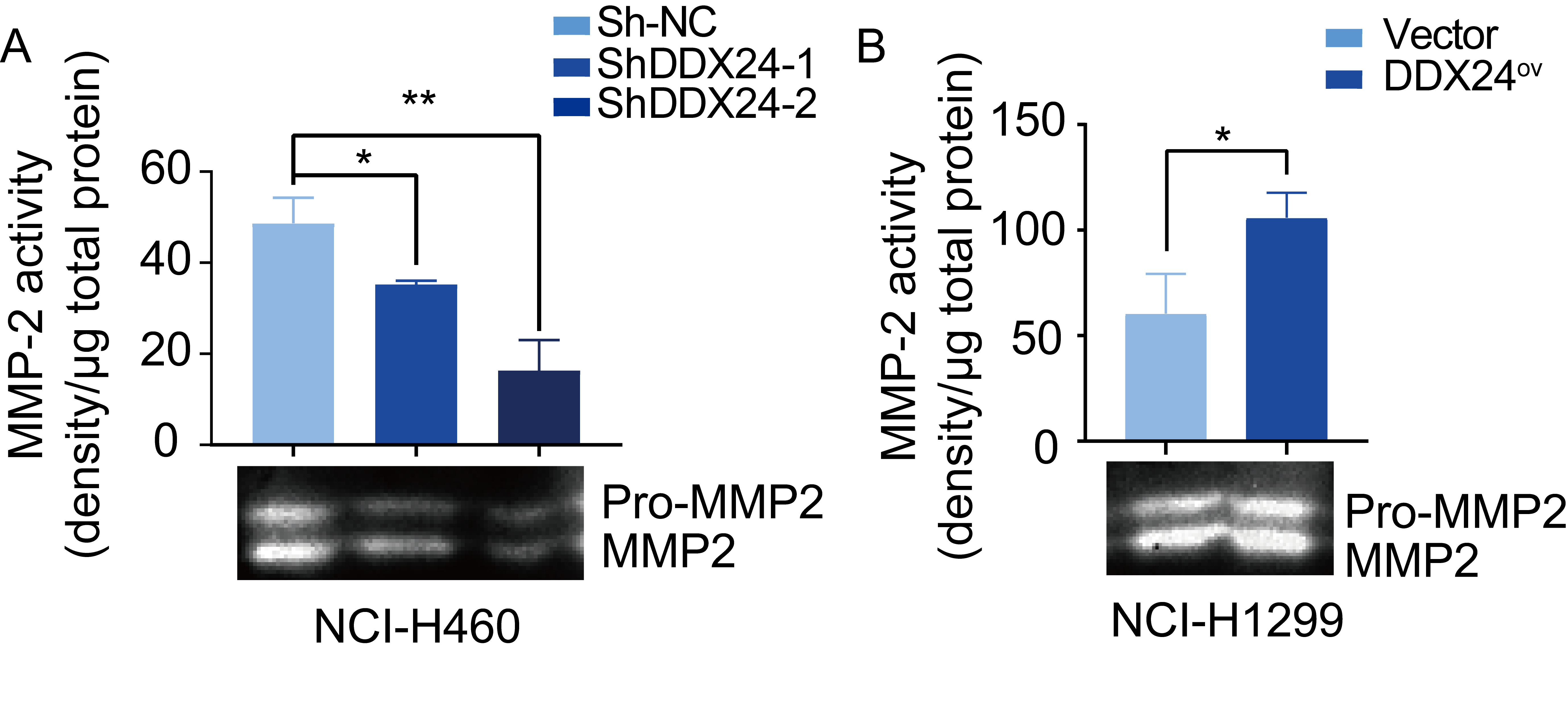

Supplement: Supplementary file 2 — Figure S2 [file CAM4-11-4513-s002.tif]

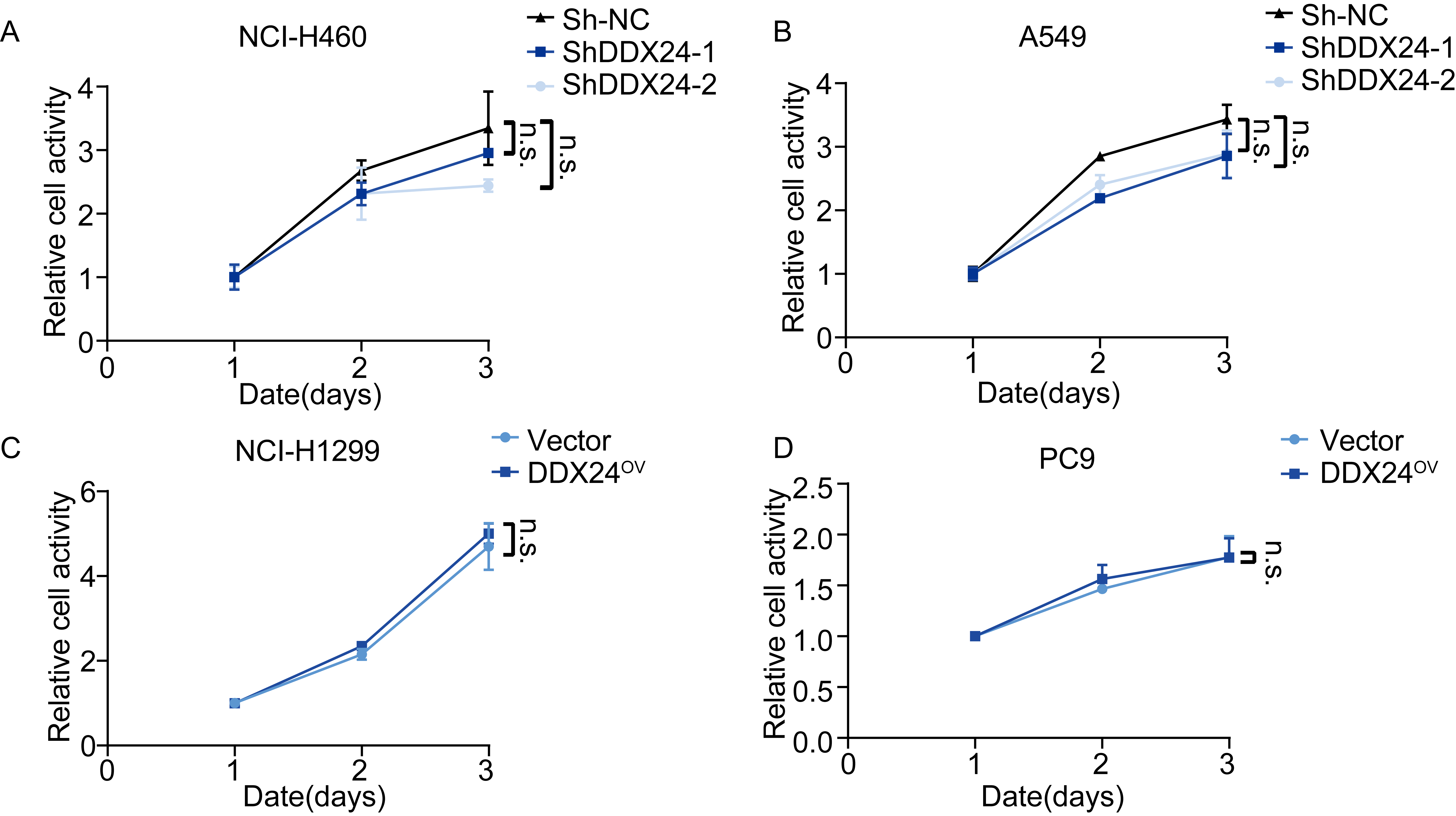

Supplement: Supplementary file 3 — Figure S3 [file CAM4-11-4513-s003.tif]
