# Supplementary material for: DDX24 promotes metastasis by regulating RPL5 in non‐small cell lung cancer
Source: Cancer Med. 2022 Jul 21;11(23):4513–25. doi: 10.1002/cam4.4835 (PMC9741967; doi:10.1002/cam4.4835)
Supplement: Supplementary file 4 — File S1 [file CAM4-11-4513-s001.docx]

SUPPLEMENTARY INFORMATION FOR:

Article Title: DDX24 Promotes Metastasis by Regulating RPL5 in Non-small Cell Lung Cancer

Journal Name: Cancer Medicine

Authors: Xinyan Hu; Fangfang Li; Yulan Zhou; Hairun Gan; Tiancheng Wang; Luting Li; Haoyu Long; Bing Li; Pengfei Pang

Corresponding Author:

Pengfei Pang (Main Corresponding Author)

E-mail address: pflb@sina.com

Department of Interventional Medicine, The Fifth Affiliated Hospital, Sun Yat-sen University, Zhuhai, 519000 P. R. China

Bing Li

E-mail address: pangpf@mail.sysu.edu.cn

Department of Ophthalmology, The Fifth Affiliated Hospital, Sun Yat-sen University, Zhuhai, 519000 P. R. China

This document contains the following:

Additional Tables of Content

Page 1-8: Supplementary Table 1. The summary of protein interacting with DDX24 protein identified by mass spectrometry analysis

Supplementary Table 1

| ProteinID | GeneName |
| --- | --- |
| P35908 | *KRT2* |
| H6VRF8 | *KRT1* |
| P02533 | *KRT14* |
| P35527 | *KRT9* |
| P08779 | *KRT16* |
| B4DRR0 |  |
| Q8NI62 | *OK/KNS-cl.6* |
| B4DN72 |  |
| B2R4M6 |  |
| P13645 | *KRT10* |
| P13647 | *KRT5* |
| F5H265 | *UBC* |
| B4DKV4 |  |
| Q04695 | *KRT17* |
| Q6NSF2 | *RPLP0* |
| O76009 | *KRT33A* |
| F6KPG5 |  |
| P31151 | *S100A7* |
| H0YMD0 | *ANXA2* |
| P78386 | *KRT85* |
| A0A2R8Y6J3 | *RPL5* |
| Q5TCU3 | *TPM2* |
| P01040 | *CSTA* |
| G3V529 | *DDX24* |
| B4E335 |  |
| B4E3A4 |  |
| A0A140TA69 | *KRT34* |
| V9HW25 | *HEL-S-273* |
| D6RCN3 | *ANXA5* |
| F8WD59 | *RPSA* |
| P78385 | *KRT83* |
| A0A087WWU8 | *TPM3* |
| Q14525 | *KRT33B* |
| A0A5C2FUL3 |  |
| Q5T8U3 | *RPL7A* |
| Q15323 | *KRT31* |
| P81605 | *DCD* |
| Q8N1N4 | *KRT78* |
| P31944 | *CASP14* |
| F5H7S3 | *TPM1* |
| F5H1V1 | *ARF3* |
| P61247 | *RPS3A* |
| A0A0G2JQH2 | *RPS18* |
| A0A0K0K1I0 | *HEL-S-265* |
| Q0QET7 | *GAPDH* |
| P67936 | *TPM4* |
| Q86YZ3 | *HRNR* |
| K7ERE3 | *KRT13* |
| P08670 | *VIM* |
| Q14532 | *KRT32* |
| A0A024R1X8 | *JUP* |
| P22531 | *SPRR2E* |
| Q9BYR6 | *KRTAP3-3* |
| O95678 | *KRT75* |
| P18124 | *RPL7* |
| Q45KI0 | *PRSS1* |
| P12273 | *PIP* |
| H0YAW3 | *DECR1* |
| B2R4K7 |  |
| C9JB90 | *RAB6B* |
| A0A0A0MSI0 | *PRDX1* |
| H3BSP4 | *PCBP2* |
| P62753 | *RPS6* |
| A0A1W2PS15 | *TCEA1* |
| P15880 | *RPS2* |
| H0YI54 | *KRT78* |
| F8W6I7 | *HNRNPA1* |
| D6RF44 | *HNRNPD* |
| Q96IR1 | *RPS4X* |
| H3BN34 | *PKM* |
| C4AM86 | *KRT35* |
| Q5JR95 | *RPS8* |
| F8VV32 | *LYZ* |
| A0A5C2GD32 |  |
| Q01469 | *FABP5* |
| F8W1N5 | *NACA* |
| V9GZN0 |  |
| P33764 | *S100A3* |
| Q6IPH7 | *RPL14* |
| P16403 | *H1-2* |
| A0A140VJQ2 |  |
| K7EM20 | *YWHAE* |
| B4DE59 |  |
| E9PN25 | *HSPA8* |
| E9PL09 | *RPS3* |
| A0A4D5RAI5 |  |
| P05787 | *KRT8* |
| O75223 | *GGCT* |
| P62805 | *H4C1H4C2H4C3H4C4H4C5H4C6H4C8H4C9H4C11H4C12H4C13H4C14H4C15H4-16* |
| F5GY37 | *PHB2* |
| P35321 | *SPRR1A* |
| A0A1B0GW87 | *U2AF1L5* |
| B3KM80 | *NCL* |
| P16401 | *H1-5* |
| Q9BYR8 | *KRTAP3-1* |
| C9JP48 | *PPP1CB* |
| J3QSB4 | *RPL13* |
| B4DL14 |  |
| P12236 | *SLC25A6* |
| P62861 | *FAU* |
| A0A5F9ZH78 | *ARG1* |
| O76015 | *KRT38* |
| D6R9P3 | *HNRNPAB* |
| A0A5C2GKH6 |  |
| B4DF70 |  |
| P05198 | *EIF2S1* |
| H0YLR3 | *SNRPA1* |
| Q92522 | *H1-10* |
| Q02413 | *DSG1* |
| A5D904 | *RPS9* |
| Q8IWR8 |  |
| A0A6Q8PHJ6 | *HSPB1* |
| Q701L7 | *KRTHB2* |
| P62851 | *RPS25* |
| Q9NXV2 | *KCTD5* |
| Q0KKI6 |  |
| H3BUH7 | *ALDOA* |
| Q9BYG3 | *NIFK* |
| A0A087WYY5 | *PPP1CC* |
| G3V4W0 | *HNRNPC* |
| M0R0P1 | *FBL* |
| P08729 | *KRT7* |
| P15924 | *DSP* |
| E9PS23 | *CFL1* |
| E9PKZ0 | *RPL8* |
| C9JI87 | *VDAC1* |
| A0A024R952 | *PKP1* |
| B7Z3E5 |  |
| B4DJI1 |  |
| A0A024RA28 | *HNRPA2B1* |
| Q0IIN1 | *KRT77* |
| M0R210 | *RPS16* |
| C9JUP7 | *VCP* |
| F5GXG4 | *PGAM5* |
| Q9NSB2 | *KRT84* |
| Q96P68 | *OXGR1* |
| A8K0T9 |  |
| Q6KB66 | *KRT80* |
| B4DUI5 |  |
| A0A1W2PP35 | *HNRNPU* |
| P39019 | *RPS19* |
| M0R0G9 | *SNRPA* |
| P10599 | *TXN* |
| P05109 | *S100A8* |
| A0A2R8Y4U1 |  |
| B8ZZK4 | *RPL31* |
| P63244 | *RACK1* |
| Q9BYQ3 | *KRTAP9-3* |
| P02763 | *ORM1* |
| P31025 | *LCN1* |
| Q16643 | *DBN1* |
| F8WEG8 | *PRKRA* |
| C9JZ20 | *PHB* |
| B5BU24 | *YWHAB* |
| Q96P63 | *SERPINB12* |
| B4DRW1 |  |
| Q9HCY8 | *S100A14* |
| A8K9K8 |  |
| F8VUA6 | *RPL18* |
| J3KTJ3 | *RPL23* |
| Q5T749 | *KPRP* |
| B4E3E6 |  |
| A0A4D5RAB8 |  |
| P59665 | *DEFA1DEFA1B* |
| P63104 | *YWHAZ* |
| K7ERG9 | *CFD* |
| D6RE67 | *TMA16* |
| B4DIU0 |  |
| A8K9J7 |  |
| P0DOY2 | *IGLC2* |
| Q96AG4 | *LRRC59* |
| Q13747 |  |
| B7ZM39 | *KRT40* |
| B2R5H0 |  |
| E9PI21 | *HSD17B12* |
| A0A5C2GME4 |  |
| Q4ZG77 | *PSMD14* |
| B4DQ50 |  |
| Q96QR8 | *PURB* |
| Q53S41 | *YWHAQ* |
| P05141 | *SLC25A5* |
| C9JXZ7 | *RFC4* |
| Q9BYT5 | *KRTAP2-2* |
| P47929 | *LGALS7LGALS7B* |
| A0A4P8J6J1 |  |
| Q9ULW3 | *ABT1* |
| B4DUA2 |  |
| A0A0G2JMB2 | *IGHA2* |
| Q8WVV4 | *POF1B* |
| B1AK87 | *CAPZB* |
| B4DJK0 | *SRSF5* |
| Q15717 | *ELAVL1* |
| M0R3F1 | *HNRNPUL1* |
| G3XAL0 | *MDH2* |
| Q9HB00 | *DSC1* |
| H0YJM8 | *PSMB5* |
| E7EP32 | *GNB2* |
| Q3LI55 | *KRTAP11-1* |
| Q7RTS7 | *KRT74* |
| P25311 | *AZGP1* |
| B3KX15 |  |
| Q15828 | *CST6* |
| H0YKS0 | *PSMA4* |
| Q2VPJ6 | *HSP90AA1* |
| A0A3B3IS95 |  |
| B4DDU2 |  |
| B9EG90 | *TOP1* |
| B2R8R5 |  |
| E9PLA9 | *CAPRIN1* |
| Q6UWP8 | *SBSN* |
| C9JMD7 | *BCAP31* |
| H7C0K7 | *RPUSD3* |
| Q0EFA5 | *S* |
| Q7Z3N6 | *DKFZp686A01173* |
| B0YJ74 | *PSMA8* |
| K4DIB6 | *SLC25A22* |
| Q96BG6 | *ACTN4* |
| A0A1U9X8Y4 |  |
| Q9NQT5 | *EXOSC3* |
| Q8IYY2 | *KCTD2* |
| D3VVN5 | *ATXN3* |
| A0A1B0GW44 | *CTSD* |
| F8VNY5 | *CCDC59* |
| B4DHC4 |  |
| P11021 | *HSPA5* |
| Q5JXM0 | *DKFZp564C0482* |
| P28072 | *PSMB6* |
| Q8N6M0 | *OTUD6B* |
| Q2VIL4 |  |
| B4DSH1 |  |
| P25788 | *PSMA3* |
| P31947 | *SFN* |
| Q8IX02 |  |
| K7EJ01 | *AP2B1* |
| J3QRE9 | *KRT39* |
| H0Y9V2 | *UBE2B* |
| Q13867 | *BLMH* |
| Q5D862 | *FLG2* |
| Q6L8H2 | *KRTAP5-3* |
| Q96SP2 |  |
| B4E0E1 |  |
| B4DDM6 |  |
| I0B0K5 | *FLG* |
| B4DKM5 |  |
| Q6ZVX7 | *NCCRP1* |
| A0A0J9YY76 | *ESPN* |
| B4DPU3 |  |
| A8K335 |  |
| A0A2R8Y6G6 | *ENO1* |
| B4DP62 | *SLC25A1* |
| C9JZ65 | *SERPINB4* |
| Q17RP2 | *TIGD6* |
| D7RXV9 |  |
| E9PDF6 | *MYO1B* |
| Q8NHX6 | *HCC5* |
| P78345 | *RPP38* |
| F5GYR3 | *NOP2* |
| O75342 | *ALOX12B* |
| B4DF00 |  |
| Q86TW7 | *DLST* |
| A8K4Z2 |  |
| A0A5F9ZHC5 | *AIMP1* |
| B4DHT9 |  |
| C9J3L8 | *SSR1* |
| H3BQN7 | *SMG1* |
| E9PLJ2 | *CHIA* |
| Q8NDZ2 | *SIMC1* |
| Q53GG0 |  |
| B3KPZ8 |  |
| B2RAM6 |  |
| B4E395 |  |
| P0DPA2 | *VSIG8* |
| A0A024RBE8 | *SLC25A3* |
| Q53G15 |  |
| B7Z1N5 |  |
| B3KX99 |  |
| B4DWK8 |  |
| Q96GC8 | *NOP14* |
| Q12792 | *TWF1* |
| P25705 | *ATP5F1A* |
| A0A514C937 | *ZC3HAV1* |
| B3KWI4 |  |
| A8K5N5 |  |
| Q96MR6 | *CFAP57* |
| Q2M243 | *CCDC27* |
| Q12797 | *ASPH* |
| Q86SJ6 | *DSG4* |
| Q5C9Z4 | *NOM1* |
| Q9NR30 | *DDX21* |
| P35579 | *MYH9* |
| F4MH35 | *UTY* |
| Q9Y446 | *PKP3* |
| Q01780 | *EXOSC10* |
| Q96T88 | *UHRF1* |
